# Automatic Localization and Count of Agricultural Crop Pests Based on an Improved Deep Learning Pipeline

**DOI:** 10.1038/s41598-019-43171-0

**Published:** 2019-05-07

**Authors:** Weilu Li, Peng Chen, Bing Wang, Chengjun Xie

**Affiliations:** 1 School of Electrical and Information Engineering, Anhui University of Technology, 243032 Ma’anshan, Anhui China; 2 Institutes of Physical Science and Information Technology, Anhui University, 230601 Hefei, Anhui China; 3 Institute of Intelligent Machines, Chinese Academy of Sciences, 230031 Hefei, Anhui China

**Keywords:** Computational science, Computer science

## Abstract

Insect pests are known to be a major cause of damage to agricultural crops. This paper proposed a deep learning-based pipeline for localization and counting of agricultural pests in images by self-learning saliency feature maps. Our method integrates a convolutional neural network (CNN) of ZF (Zeiler and Fergus model) and a region proposal network (RPN) with Non-Maximum Suppression (NMS) to remove overlapping detections. First, the convolutional layers in ZF Net, without average pooling layer and *fc* layers, were used to compute feature maps of images, which can better retain the original pixel information through smaller convolution kernels. Then, several critical parameters of the method were optimized, including the output size, score threshold, NMS threshold, and so on. To demonstrate the practical applications of our method, different feature extraction networks were explored, including AlexNet, ResNet and ZF Net. Finally, the model trained on smaller multi-scale images was tested on original large images. Experimental results showed that our method achieved a precision of 0.93 with a miss rate of 0.10. Moreover, our model achieved a mean Accuracy Precision (mAP) of 0.885.

## Introduction

In recent years, detecting pests in crops-fields has become a hot topic. More and more farmers, governments and researchers have focused on pest detection, which is regarded as a useful tool in precision agriculture. Automatically monitoring the number of crop pests across large crop area has evolved into one of the important means for managing and optimizing agricultural resources^1–4^. There is a variety of wheat pests that seriously affect wheat growth, among which wheat mite is common and much dangerous. Thus, visual mining of crop pests is an important research objective, especially in forecasting of the wheat pests and diseases.

Because wheat mites are very small and their count is hard to assess, traditional methods based on visual estimation cannot investigate wheat mites accurately. The rapid development of image processing techniques has paved a new way for pest recognition. Therefore, collecting images by camera and further counting the number of pests in images using advanced image techniques has become the direction of intelligent agricultural systems.

Currently, there are two types of methods in the image-based detection of insects. One method is based on traditional image processing and machine learning algorithms; while the other is based on deep learning. A detection system based on traditional machine learning algorithms is composed of three sequential phases: image capture and annotation^5^, feature extraction and object detection. However, to design an extractor of features, such as color^6,7^, shape and texture^8,9^, sparse coding and multi-kernel learning^10,11^ has to be used. Albeit, the traditional machine-learning techniques require precision engineering and considerable domain expertise.

Not only an effective feature extractor, but also a classifier to object detection is needed. Support vector machine (SVM) is the most common choice of many researchers, which is a supervised learning method that generates input-output mapping functions from a set of labeled training data. Many authors adopted SVM classifier or bag-of-words approach to classify rice crop pests^4^. They developed a pest recognition system based on image processing techniques, including bio-inspired filtering, LCP algorithm and SVM^12^. The experimental results showed that representations learned by the recognition system often obtained a better performance than hand-designed representations.

Since 2016, deep learning technologies have been successfully applied in many research fields, such as image processing, speech recognition and so on. Many researchers have made use of deep neural networks to automatically extract features from original images and learn low-level to high-level features represented by the images. The key aspect of deep learning is that features are learned from images using a multi-layer neural network, such as ConvNet or ResNet^13^. For example, Ding and Taylor developed a ConvNet-based pest detection method with a novel pose-estimation-dependence and automated identification, which can be applied in recognizing pest species^14^. They proposed an improved pyramidal stacked denoising auto-encoder (IpSDAE) architecture to build a deep neural network for moth identification to achieve a good identification accuracy^14^. Wang *et al*. developed a novel method to identify the pollution of crop pests by combining a near infrared recognition device with the technique of Principal Component Analysis^3^.

**Figure 1:**
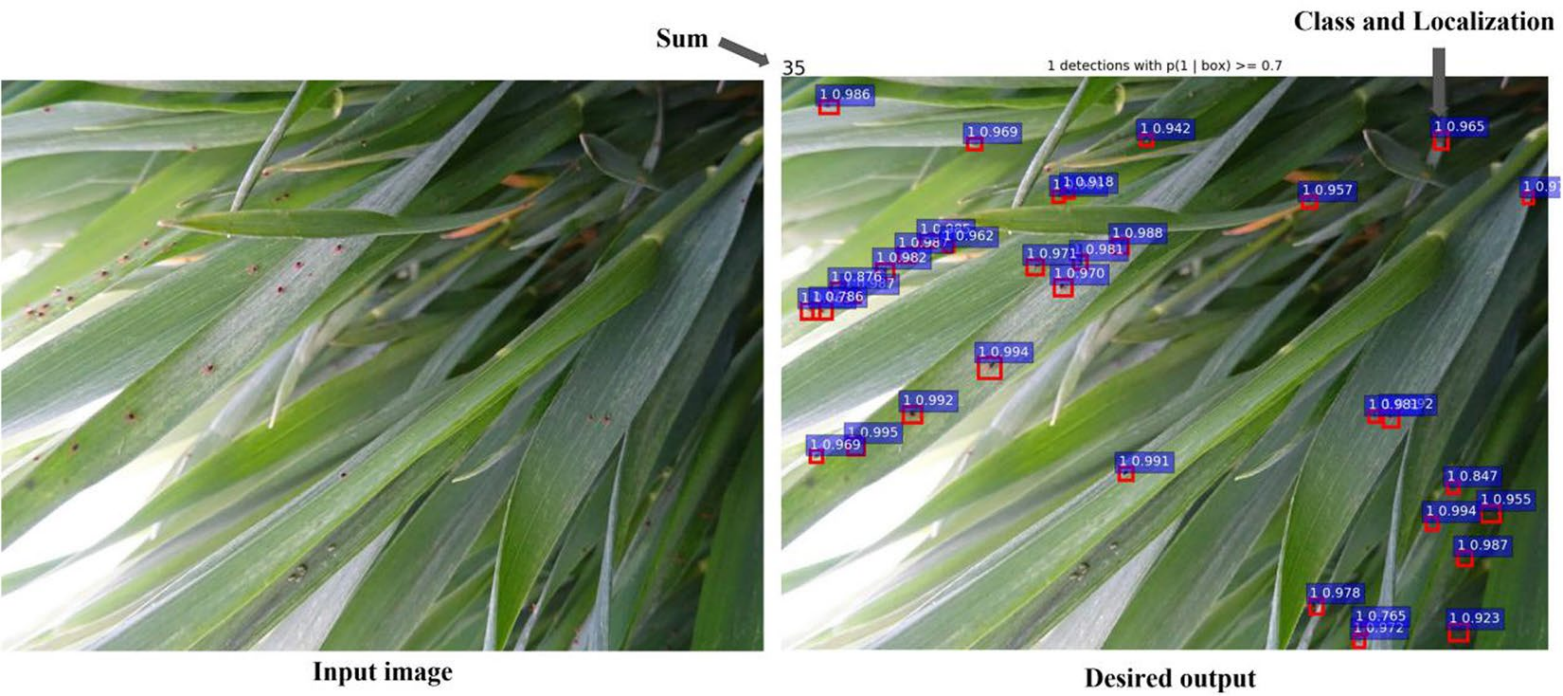
Classification and localization of wheat mites. Our method aims to detect both the classification and localization of wheat mites. At the same time, this method outputs the total number of wheat mites in the original images. The left graph shows the case of original image, while the right one illustrates classification and localization of wheat mites. Here, the location of wheat mite in the image is shown as a red bounding box containing the identified wheat mites, the possibility of detected bounding box is shown in blue box, and the total number of 35 detected wheat mites is also shown in the right graph.

Although these methods yielded good results, few methods have been developed for recognizing small pests. This paper proposes a deep learning-based system to identify and count a very small pest, wheat mites. First, original images were transformed into multi-scale images and further input into a stack of convolution layers called fully convolutional network (FCN), to extract efficient features. Second, specialized convolutional layers were added after the last convolutional layer of FCN to construct a set of positive-sensitive score maps. Third, the outputs of the last convolutional layer in FCN were input into an RPN that produced region proposals, which were then applied on the positive-sensitive score maps. Finally, these region proposals and score maps were input into PoI Pooling to output classifications and target boxes. Figure 1 illustrates the result cases of our deep learning-based system, which is capable of estimating the number and class of pests.

## Methodology

### Datasets

Our database contains 84 images with 1440 × 1080 pixels each, taken by digital cameras to capture wheat mites at any angle in wheat fields. The original database is separated into two sets: training dataset (64 images) and test dataset (20 images). The images of the training dataset were transformed into two levels: single-scale images and multi-scale images. Next, each of the original images from the datasets was cropped into smaller images of various sizes (e.g., 150 × 250, 240 × 400, 300 × 500 and 600 × 1000), where images cropped by one multi-scale have the same aspect ratio (e.g., 3: 5). Finally, as shown in Table 1, 850 and 378 smaller images that were identifiable by naked eye were generated from the training and test datasets, respectively.

**Table 1:** The distribution of the training and test datasets.

	Transformation	Pixel	Size of training dataset	Size of test dataset
Original images		1440 × 1080	64	20 (Dataset B)
Transformed images	Cropped images with multi-scales	600 × 1000	98	37
		300 × 500	178	78
		240 × 400	243	111
		150 × 250	331	152
		Total	850	378
	Flip transformation	600 × 1000	98	
		300 × 500	178	
		240 × 400	243	
		150 × 250	331	
		Total	850	
	Noise transformation	600 × 1000	196 × 2	
		300 × 500	356 × 2	
		240 × 400	486 ×	
		150 × 250	662×2	
	Total		3400	378 (Dataset A)

### Data augmentation

For deep learning methods, typically, larger the dataset, the better is the model’s performance. Since there were only 850 images in our training dataset, which cannot make full use of the power of deep learning models, a data augmentation technique was adopted to increase the size of the dataset. Moreover, each image was transformed by flipping across the horizontal-axes and by adding salt-and-pepper noises. As a result, three times as many as images, in the form of augmented images, were obtained containing the original small images. As a result, the transformed training dataset comprised of 3,400 transformed images, while the transformed test dataset had 378 images, which is regarded as “Dataset A”. However, the original 20 images comprised the test dataset (see Table 1) and were regarded as “Dataset B” in this work.

### Caffe

Caffe is a deep learning framework that contains an expressive architecture for encouraging application and innovation, extensible code for fostering active development and the speed. Caffe has been successfully applied in many research experiments and industry deployment, such as in academic research projects, start up prototypes. Caffe has also been used in large-scale industrial applications in vision, speech and multimedia. In this work, we used the framework to implement and train different ResNet and ConvNet models. Caffe has many predefined neural network layers and packages that enabled us to run deep learning algorithms on GPUs.

### Deep convolutional neural network

#### Overall Architecture

**Figure 2:**
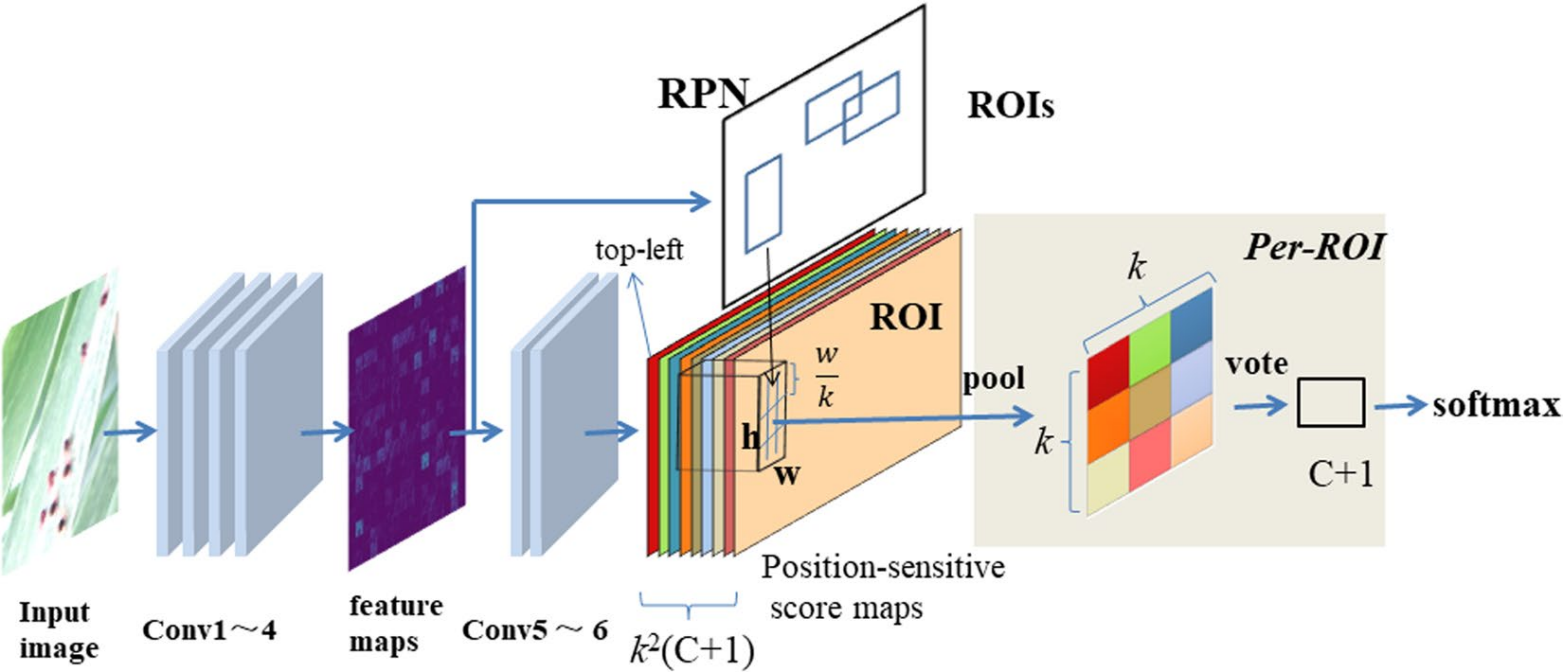
Flowchart of our deep learning-based method. The method consists of four convolutional layers (1~4) for extracting feature maps, two convolutional layers (5~6), a RPN containing *k*^2^(*C* + 1) ROIs to detect position-sensitive score maps and make final results, as well a softmax layer to output classifications and localizations.

**Figure 3:**
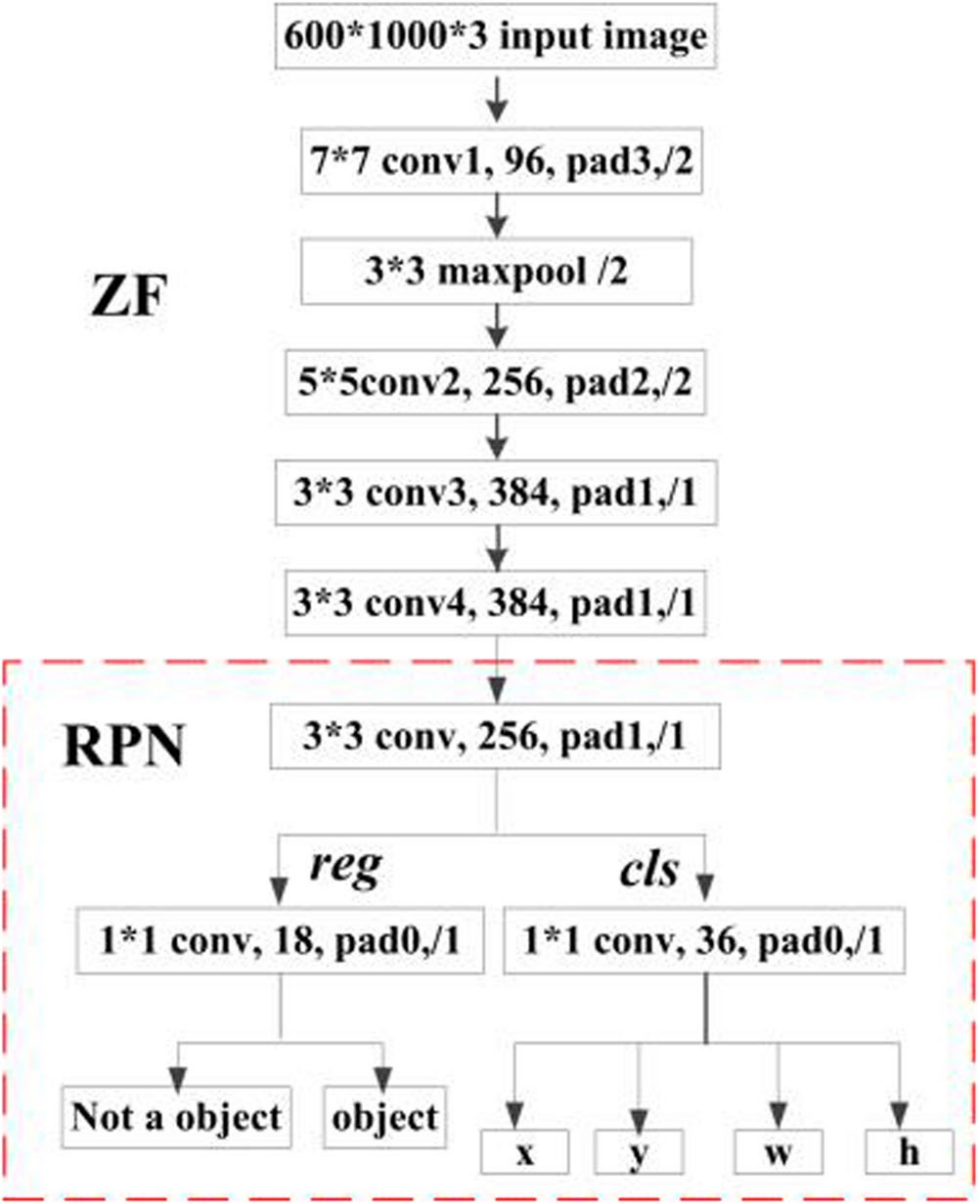
Architecture of ZF and RPN. The top part shows the adopted structure of ZF, which consists of four convolutional layers, conv1 to conv4, and a maxpool layer. The bottom part shows the adopted structure of RPN, which contains a convolutional layer and two output layers, *reg* and *cls*.

Our method consists of ZF (Zeiler and Fergus model^15^) and RPN (Region Proposal network) networks. Figure 2 illustrates the overall architecture of these networks, and Fig. 3 shows the individual architecture of ZF and RPN. The ZF network adopted a part of the Zeiler and Fergus model (ZF model)^15^, pre-trained on ImageNet. ZF is composed of 5 sharable convolutional layers and 2 4096-d *fc* layers. Removing the average pooling layer and the two *fc* layers, only four convolutional layers were retained to compute the feature maps, from conv1 to conv4, as shown in Fig. 2. The modified ZF network can be thought of as a self-learning progression of local image features, from low- to mid- to high-level. The last convolutional layer, conv5, was 256-d. Also, a randomly initialized 1 × 1 convolutional layer, conv6, was attached to increase the dimension to 1024-d. Then, the *k*^2^*(C* + *1)*-channel convolutional layer was applied to compute Position-sensitive score maps, where *k* denotes the dimension of the unified feature map, and *C* is the number of classes. For each Region of Interest (RoI), the *k* × *k* feature map can be obtained, which pools from the Position-sensitive score maps.

#### Region proposal network (RPN)

RPN is used for generating region proposals in detectors such as Faster R-CNN (Regions with CNN) and R-FCN (Region-based Fully Convolutional Networks). After obtaining feature map from the conv4 of the pre-trained model, region proposals could be implemented over the feature map. A 3 × 3 window was made to slid over the feature map, and each sliding window was mapped to a 256-dimensional sub-feature map. Afterwards, these sub-feature maps were fed into the following two fully convolutional layers: box-regression layer (*reg*) and box-classification layer (*cls*). In each sliding window, *k* region proposals and the corresponding *k* detection boxes were obtained. Thus, *2k* scores were generated in the *cls* layer and *4k* outputs in the *reg* layer. Since each proposal is associated with four coordinates, each proposal is centered at the sliding window in question and associated with a scale and aspect ratio. Three scales with box areas, 4, 8 and 16, as well as three aspect ratios of height-to-width, 0.8, 1 and 1.5, were used, respectively. As a result, 3 × 3 = 9 anchor boxes for each sliding position were yielded. For a convolutional feature map with size *W* × *H*, there were (*W* × *H* × *k*) anchors in total.

The anchors were then input into the two fully connected layers, namely the classification layer (*L*_*cls*_)and the regression layer (*L*_*reg*_). *L*_*reg*_ was used to predict the proposal position of the anchor, i.e., coordinates *x* and *y*, as well as the width *w* and the height *h*, while *L*_*cls*_ was used to identify the proposal as a wheat mite. Finally, according to a region proposal score, the top 200 region proposals were selected as an input to the classifier for object detection.

In addition, the loss function used in this network follows the multi-task loss to minimize the objective function, as defined in Faster R-CNN^16^. The loss function is stated as follows:

1 $$L({p}_{i},{t}_{i})=\frac{1}{{N}_{cls}}\,\sum _{i}\,{L}_{cls}({p}_{i},{p}_{i}^{\ast })+\lambda \frac{1}{{N}_{reg}}\,\sum _{i}\,{p}_{i}^{\ast }{L}_{reg}({t}_{i},{t}_{i}^{\ast }),$$

where *i* is the index of an anchor in a minibatch, and *p*_*i*_ is the prediction probability of anchor *i* being an object. The ground-truth label ${p}_{i}^{\ast }$ is 1 if the anchor is positive and 0 if the anchor is negative. Moreover, *t*_*i*_ is a vector representing the four parameterized coordinates. The classification loss *L*_*cls*_ is the log loss over two classes (object or not object), and *L*_*reg*_ is the regression loss.

#### Position-sensitive score maps

To explicitly encode position information for each RoI, the rectangle of each RoI can be divided into *k* × *k* bins using a regular grid. For an RoI rectangle with size *w* × *h*, a bin was set to the size ≈ $(\frac{w}{k}\times \frac{h}{k})$^17,18^. In our method, the last convolutional layer of the ZF Net was constructed to produce *k*^2^ score maps for each category of pest objects. Inside the (*i*, *j*)-th bin (0 ≤ *i*, *j* ≤ *k* − 1), a positive-sensitive RoI pooling operation, which follows the Region-based Fully Convolutional Network (R-FCN) that pools only over the (*i*, *j*)-th score map, is defined as follows:

2 $${r}_{c}(i,j|\theta )=\sum _{(x,y)\in bin(i,j)}\,{Z}_{i,j,c}(x+{x}_{0},y+{y}_{0})/n.$$

Here *r*_*c*_ (*i*, *j*) is the pooled response in the (*i*, *j*)-th bin for the *c*-th class, *Z*_*i*,*j*,*c*_ is a score-map out of *k*^2^ (*C* + 1) score-maps, (*x*_0_, *y*_0_) denotes the top-left corner of an RoI, *n* is the number of pixels in the bin and *θ* denotes all learnable parameters of the network.

#### Non-Maximum Suppression

Non-Maximum Suppression (NMS) is usually used object detection, whose main purpose is to remove overlapping detections by ignoring smaller overlapping bounding boxes and returning those with larger overlaps. In this work, the NMS was adapted to iterative bounding box regression in the RPN Net, at the last test stage. The RPN Net could generate approximately 20,000 proposals. The proposals were then ranked, and approximately 6,000 of top scoring proposals were retained when the score threshold was set as 0.7. Only 200 of the retained top scoring boxes were kept and input to the RoI Net. Finally, these proposals were passed through the *reg* and the *cls* layers, which output the classification and regression scores for each proposal.

**Figure 4:**
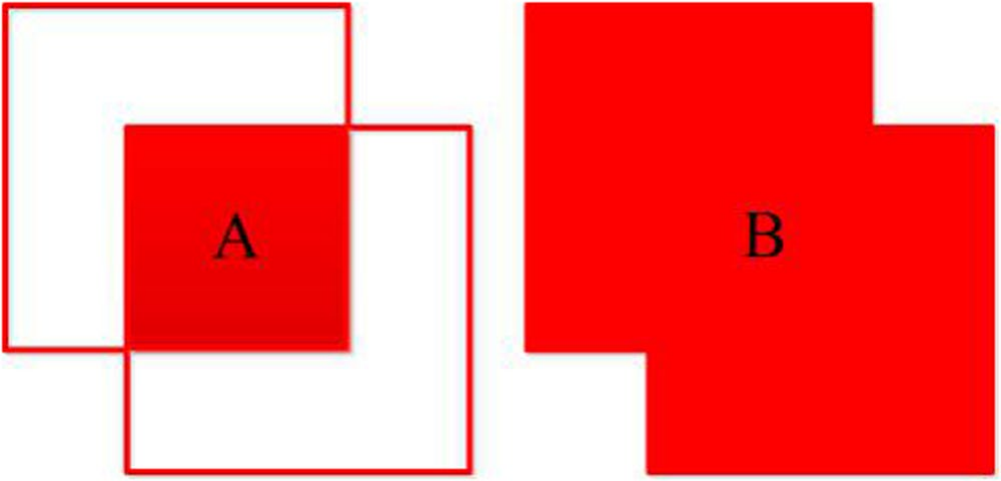
The IoU between two detection boxes. Here, (**A**) denotes the area of overlap between the two boxes, while (**B**) is the area of union of the two boxes. Therefore, the IoU is the ratio of (**A** to **B**).

In the test stage, many high-score detection boxes were output, whose overlapping detection boxes were to be kept or removed according to the Intersection over Union (IoU) ratio^16^ (see Fig. 4). First, these overlapping detection boxes, with scores greater than the score threshold, were kept if the IoU was less than the NMS threshold. Otherwise, they were removed by ignoring the overlapping bounding boxes with smaller scores and returned only those with larger scores as the final detections. The IoU was defined as follows:

3 $$IoU=\frac{Area\,of\,Overlap\,(A)}{Area\,of\,Union\,(B)}.$$

#### Optimization

Each input image was preprocessed as 600 × 1000 pixels uniformly. All the new layers of network were randomly initialized, and their weights were initialized from a zero-mean Gaussian distribution with standard deviation 0.01, which were then updated by Adam^19^, a method for efficient stochastic optimization. All the other layers were initialized by pretraining a model for ImageNet classification. All the layers of ZF net besides the conv1 layer were fine-tuned. The learning rate was initially 0.001 and was successively decreased by a factor of 10 during 2,500 step sizes, each of which consisted of 5,000 iterations. The momentum was set as 0.9 and the weight decay as 0.0005. The implementation was based on Caffe. The model was trained on a single NVIDIA GTX1080 4 GB GPU equipped on a desktop computer with an Intel i7 CPU and 16 GB of memory.

### Evaluation methods

**Figure 5:**
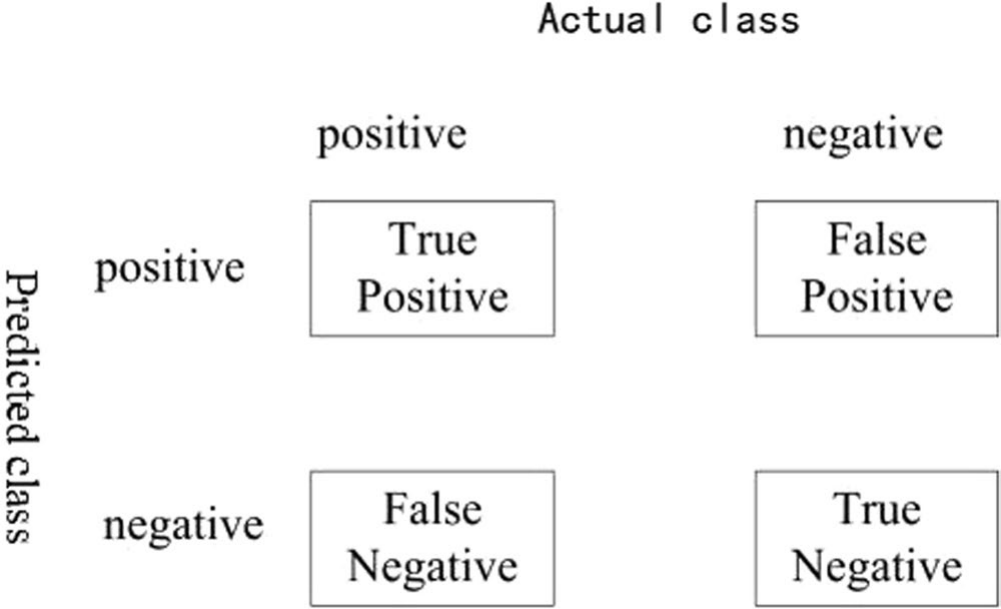
Definitions of true positive, false positive, false negative and true negative. For example, “False Positive” means that one actual negative instance was predicted to be positive by one method.

Since pest detection in agriculture is still a relatively “niche” field in computer vision, no standard evaluation protocol is defined. This paper adopted the evaluation metrics from the literature^20^ containing the statistics of true positives, true negatives, false positives and false negatives, which are illustrated in Fig. 5. The metrics were used to test if an image contained any mites. Instead of simply calculating the Mean Average Precision (mAP) of the predictions, the precision, recall, miss rate and F1 score were used as the four main evaluation metrics, defined as follows:

4 $$\begin{array}{rcl}{\rm{\Pr }}ecision & = & \frac{True\,positives}{True\,positives+False\,positives}\\ {\rm{Re}}call & = & \frac{True\,positives}{True\,positives+False\,negatives}\\ Miss\,Rate & = & 1-{\rm{Re}}\,call\\ F1\,score & = & \frac{2\times {\rm{\Pr }}ecision\times {\rm{Re}}call}{{\rm{\Pr }}ecision+{\rm{Re}}call}.\end{array}$$

### Accession codes

The source code for the methods are accessible at http://deeplearner.ahu.edu.cn/web/zfPest.htm.

## Results

In this work, the original images were taken from crop-fields by agricultural experts. There are only 64 original training images with pixels 1440 × 1080 and 20 original test images. To extend the dataset, data augmentation was adopted, leading to 3400 images for the training dataset and 378 for the test dataset. This paper adopted a simple model, i.e., pre-training deep learning model. The model used data augmentation to avoid the over-fitting problem. Our model was tested on the original images (20 images) and also on the transformed small images (378 ones, see Table 1). The experimental results showed that no over-fitting was observed in the test experiments.

### Comparisons with other feature extraction networks

The depth of a network greatly impacts the performance and training speed of the network. In this work, different feature extraction networks were investigated, such as ZFnet^15^, Alexnet^21^ and ResNet^22^, on different data types. These three networks are variations of convolutional neural networks. Since wheat mites contain two main features, i.e., very small wheat mites in the images, and a flat color for the foreground with a very complex background, a shallow network was developed to generate the feature maps of images and to minimize the loss of information as much as possible.

**Figure 6:**
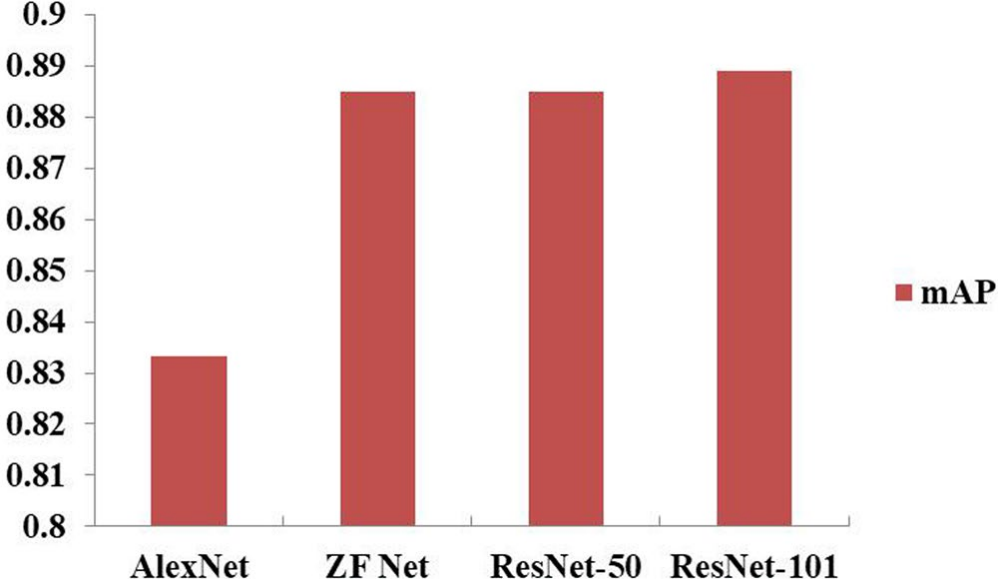
Prediction comparison of the four networks in feature extraction on the Dataset A. Here, ZF Net and ResNet performed better than AlexNet.

**Figure 7:**
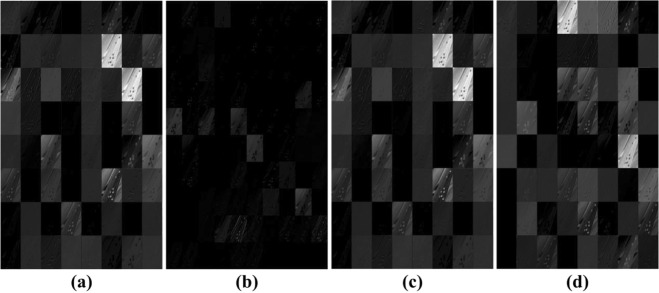
Visualization of the first convolutional layer using different feature extraction nets. (**a**) ZF Net; (**b**) AlexNet; (**c**) ResNet-50; (**d**) ResNet-101. Input images for this example were of the size 600 × 1000 pixels.

As shown in Fig. 6, it is observed that the improved ZF network achieved the best performance among the four networks, while ResNet-50 was the second best. Here, ResNet-50 and ResNet-101 have the same structure of shortcut connections, which can be used in learning residual functions. Since network information is always passed through different layers, the difference between ResNet-50 and ResNet-101 is in the number of layers. In this work, shortcut connections were also applied to our models, obtaining good results (mAP: ResNet-50: 0.885; ResNet-101: 0.889). It was also observed that ResNet-101 contains a deeper network, but only achieved an improvement of 0.4% over ResNet-50. AlexNet and ZFNet consisted of the same number of convolutional layers, but contained different filter size in the conv1 layer. ZF Net, using a smaller size of convolution kernels, saved rawer pixel information, and thus more obvious edges than AlexNet. It can be observed from Fig. 7 that the loss of pixel information for AlexNet was significant. Moreover, ZF Net had fewer parameters than ResNet; thus, ZF Net could reduce computation burden in the training process. All in all, ZF Net was regarded as a good choice in generating feature maps for our dataset.

### The roles of thresholds on counting the results

In order to show the power of our method in reducing the losses between the ground-truth and the estimated results, and in decreasing the presence of false positives in the final predictions, experiments with score threshold and NMS threshold were investigated.

**Figure 8:**
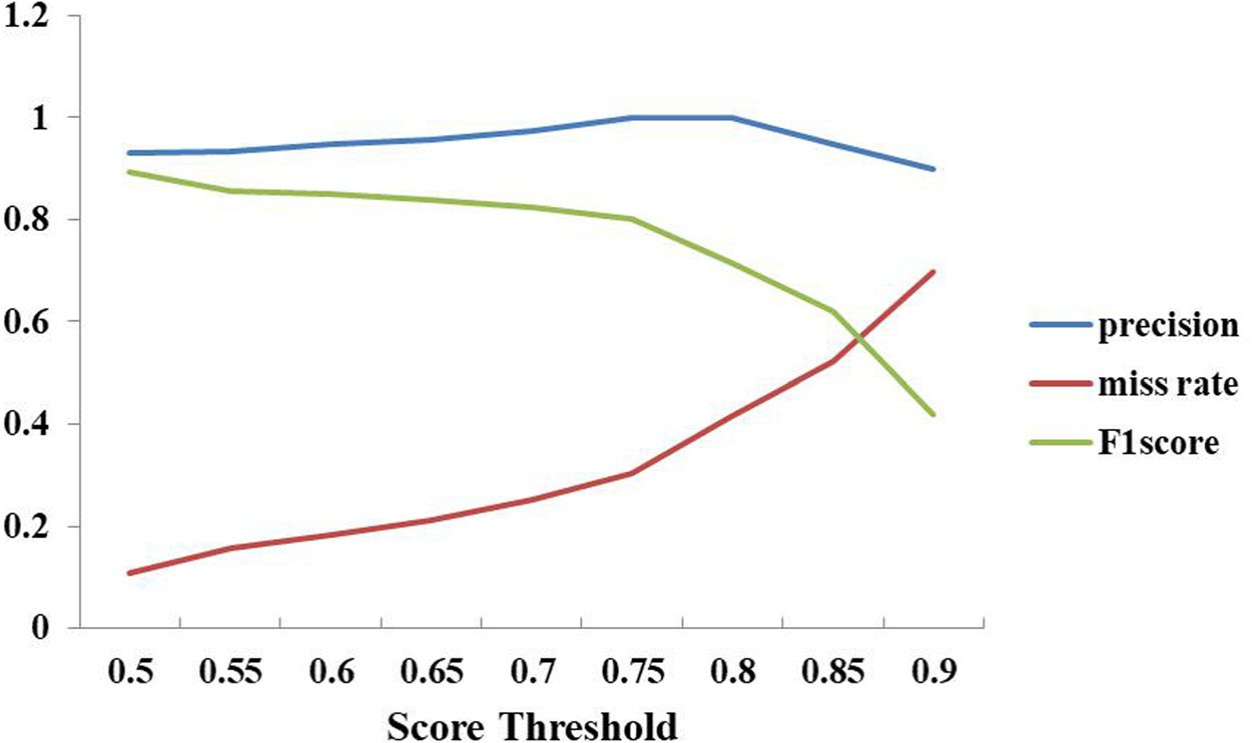
Performance of Precision, F1 score and miss rate under different score thresholds on the Dataset B. Score threshold is 0.7.

First, experiments with score thresholds, *sth*, from 0.6 to 0.9, with a stepsize of 0.05, were implemented on the Dataset B, and the detection boxes with scores larger than the threshold were visualized. A large *sth* means that the detection boxes with scores less than a given *sth* will be ignored, while the larger values will indicate presence of wheat mites. The performance on counting the number of wheat mites was calculated in terms of the precision, F1 score and miss rate, as illustrated in Fig. 8, where the model achieved maximal values of precision. The model also achieved an F1 maximal score at a score threshold of 0.6, where over 90% of counting results met the above restrictions and achieved the maximal miss rate.

**Figure 9:**
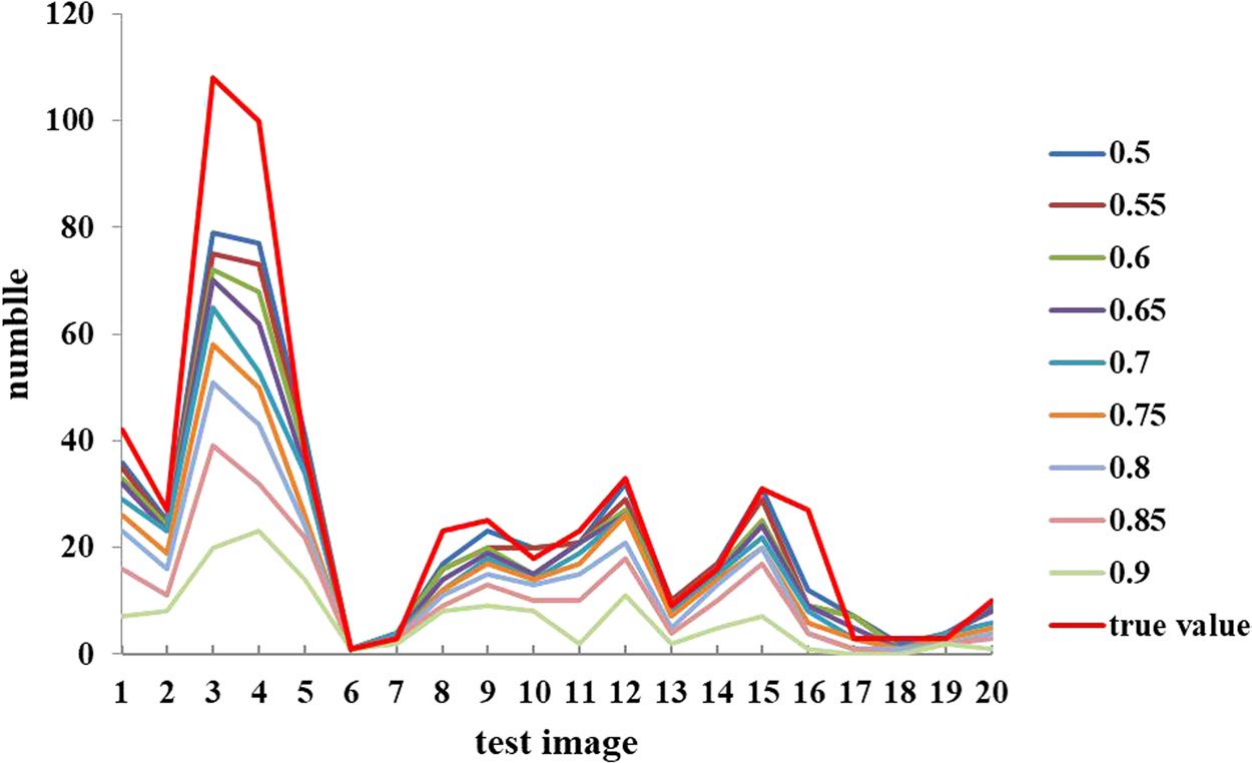
Comparison of the counting results under different score thresholds, sth. All the images for this experiment are from the Dataset B sampled with 1440 × 1080 pixels.

**Figure 10:**
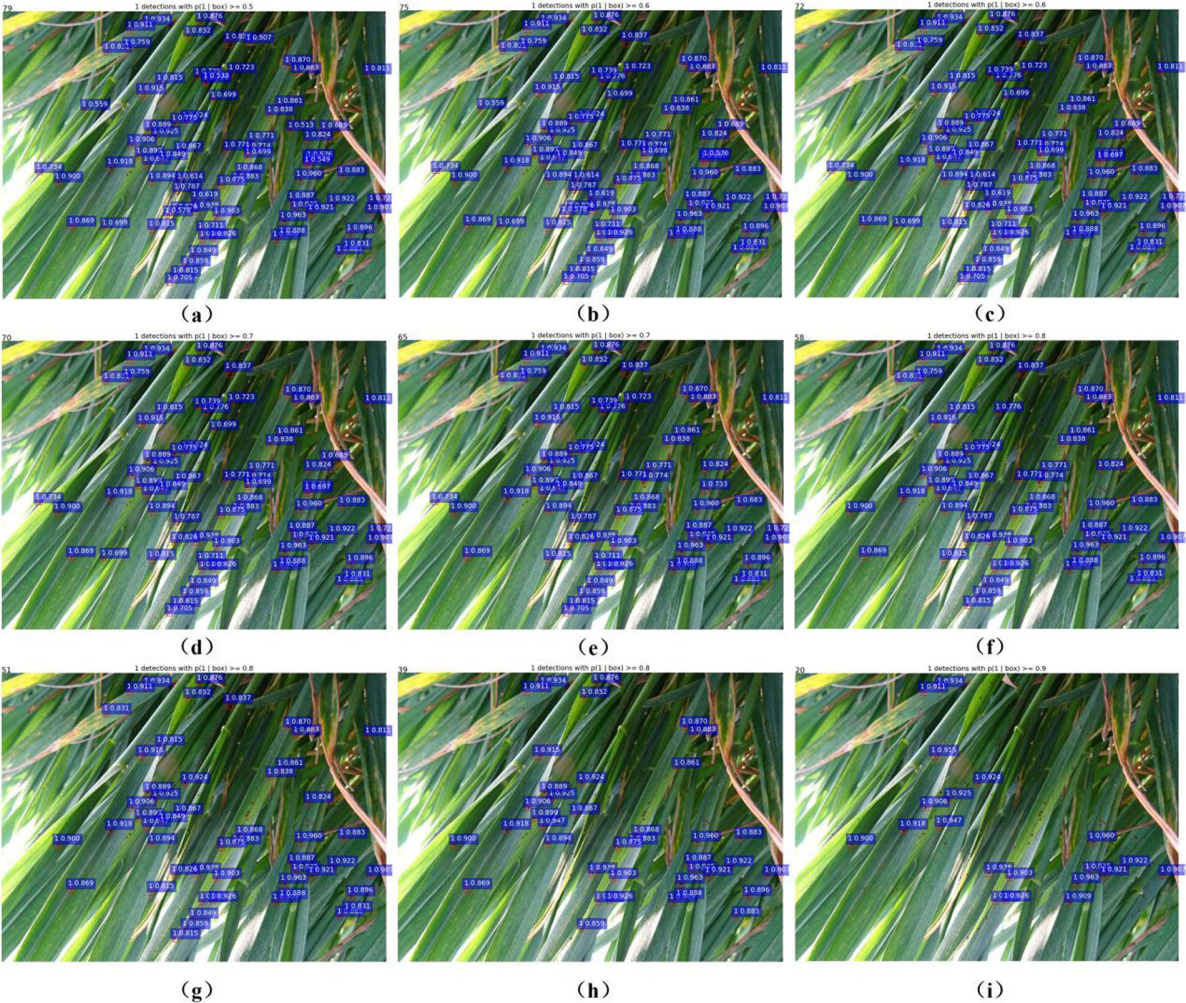
Case studies of a visual comparison of localization and counting results under different score thresholds, *sth*. (**a**) *sth* = 0.5, (**b**) sth = 0.55, (**c**) *sth* = 0.6, (**d**) *sth* = 0.65, (**e**) *sth* = 0.7, (**f**) *sth* = 0.75, (**g**) *sth* = 0.8, (**h**) *sth* = 0.85 and (**i**) *sth* = 0.9. NMS threshold is 0.5. All the images are from the dataset B stored with 1440 × 1080 pixels.

In Fig. 8, it can be observed that the precision curve becomes flat during 0.5 ≤ *sth* ≤ 0.8 and then starts to decrease when *sth* > 0.8. On the contrary, the miss rate increases all the time. While *sth* increases from 0.5 to 0.9, the number of true positives and false negatives decreases. Moreover, the number of false positives gradually declines to 0 when *sth* = 0.75, which can also be observed from the counting performance in Fig. 9. In general, *sth* = 0.5 is a relatively good choice for score threshold, given the tradeoffs among precision, F1 score and miss rate. The experimental results showed that our model can successfully detect the wheat mites of original images with 1440 × 1080 pixels (see Figs 9 and 10).

**Figure 11:**
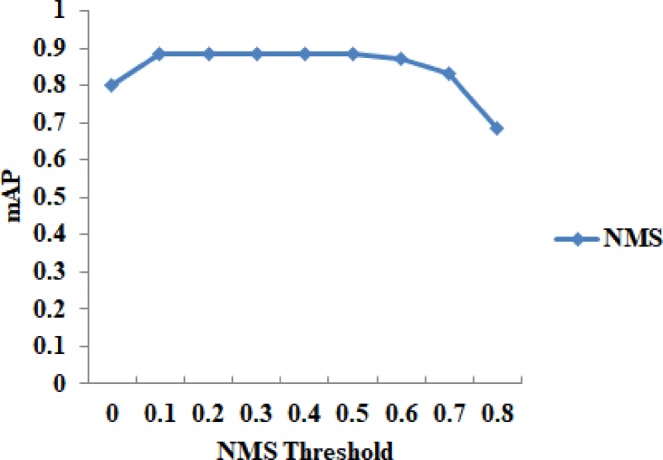
The mAP under different NMS thresholds on the dataset A.

**Figure 12:**
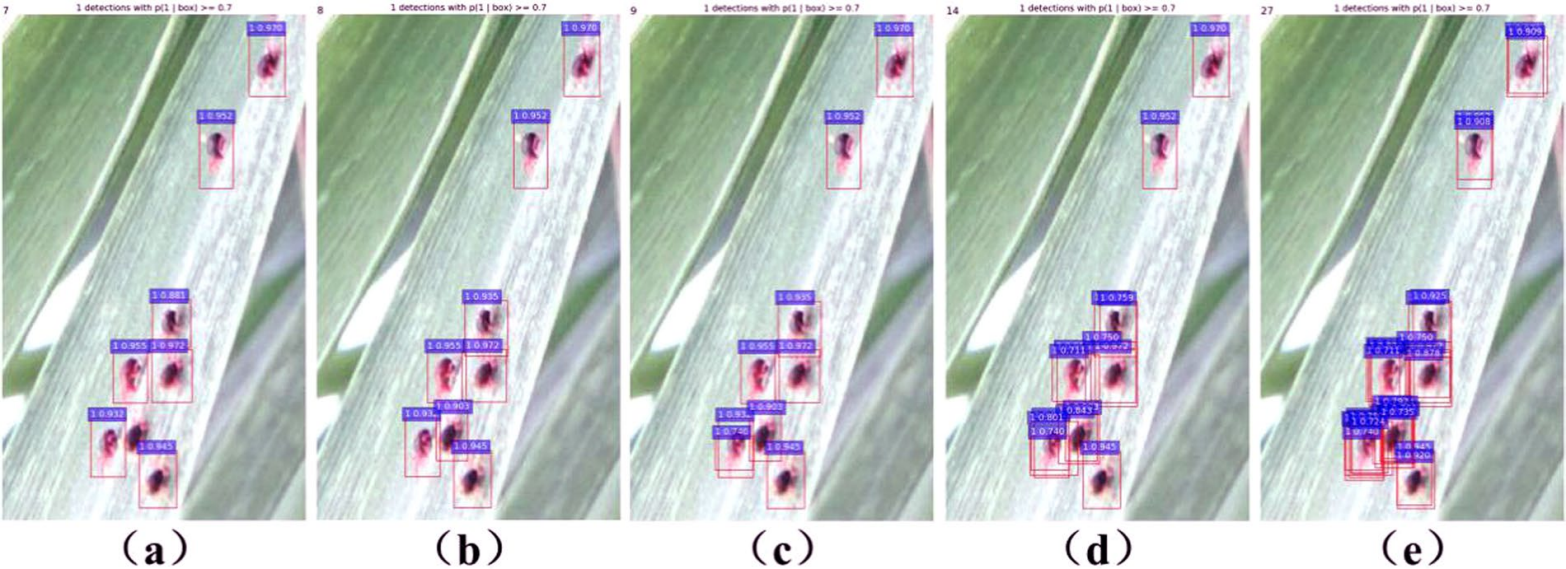
An example of the visual comparison of localization and counting results under different NMS thresholds, *nth*. (**a**) *nth* = 0. (**b**) *nth* = 0.1, 0.2, 0.3, 0.4, 0.5. (**c**) *nth* = 0.6. (**d**) *nth* = 0.7. (**e**) *nth* = 0.8. All the images for this example are from the dataset A taken with 600 × 1000 pixels.

Moreover, models with NMS thresholds, *nth*, from 0 to 0.8, with a step size of 0.1, were investigated on the dataset A, where detected boxes with NMS scores less than the threshold were kept. The mAP curve is shown in Fig. 11. The mAP curve becomes flat when 0.1 ≤ *nth* ≤ 0.5, and it decreases when *nth* > 0.5. Here *nth* = 0 means that the overlap between detection boxes is not allowed, while a larger *nth* means that more detection boxes will be kept and the overlapping boxes will be suppressed as much as possible by setting *nth* to an appropriate value (see Fig. 12). The above analysis showed that the two parameters significantly influenced the performance of our model in counting the number of wheat mites.

### Effects of the RoI output size

**Figure 13:**
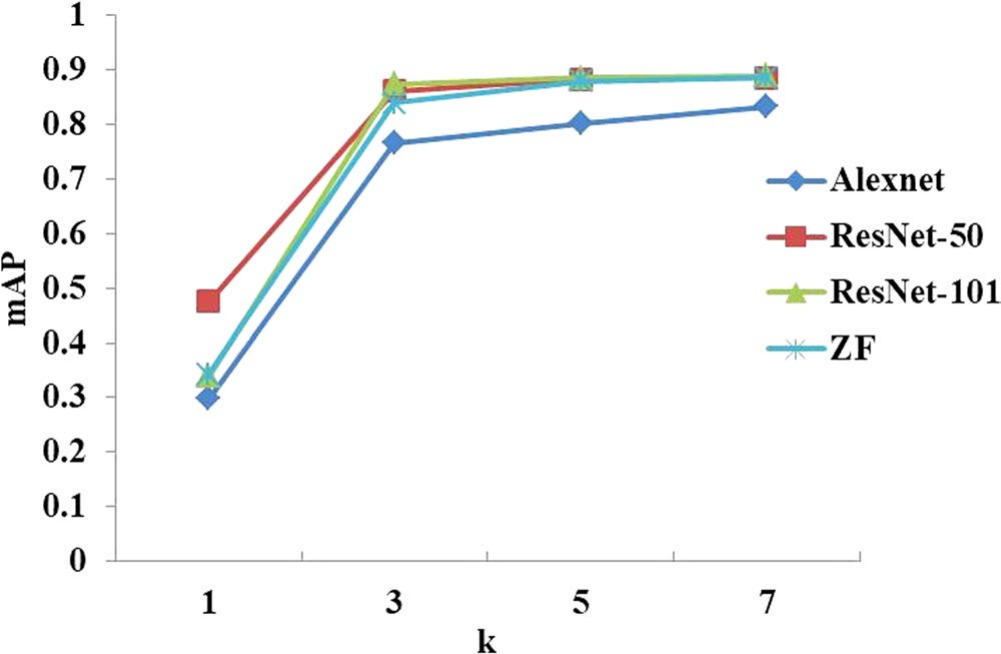
Performance comparison of the four networks with respect to different RoI output sizes.

The size, *k*, of RoI outputs is also an important factor in our model. Four other models, ZF net, ResNet-50, ResNet-101 and AlexNet, were investigated with respect of the size of RoI outputs. The performance comparison of these four models in terms of *k* is shown in Fig. 13. Setting *k* = 1 means that the role of the RoI layer was ignored, which is equivalent to global pooling within each RoI. The four models with *k* = 1 performed poorly and achieved an mAP lower than 50% because the model was unable to converge, as shown in Fig. 13. All the models conferred the biggest change in mAP when *k* increased from 1 to 3, with a small change in mAP when *k* increased from 3 to 5 or from 5 to 7. The ZF Net and ResNet-50 performed the best when *k* = *7*, where the mAP curves achieved their maximal values of approaching 90%.

### Comparison with other methods

In Table 2, we compared our model with previous methods on the Dataset A. The models Faster R-CNN with ZF and VGG_CNN_M_1024 achieved mAPs lower than 0.80. Faster R-CNN with VGG16 achieved an mAP of 0.847 under the same learning rate of 0.001. This method required half an hour to obtain the final trained-model, while the time spent by our method was no more than ten minutes. Our method yielded an mAP of 0.885, which is an improvement of 3.8% over Faster R-CNN.

**Table 2:** Comparison of our deep-learning architecture with other methods on the same dataset, where Faster R-CNN means faster region-based convolutional neural network, and SSD means single shot detector.

Method	Representation	mAP	training time
Faster R-CNN	ZF	0.764	30 min
Faster R-CNN	VGG16	0.847	40 min
Faster R-CNN	VGG_CNN_M_1024	0.754	35 min
SSD^23^	VGG16	0.874	4 h
Ours	ZF + RPN	0.885	10 min

The SSD (Single Shot MultiBox Detector) method handles the problem of object detection using separate predictors for different aspect radio detections. SSD also further applies these predictors to multiple feature maps from the later stages of a network to perform object detection at multiple scales. The method achieved an mAP of 0.874 under a learning rate of 0.0001. The training time for SSD was >4 hours, which is more than twenty times higher than that of our method to generate a trained model. In our model the objects’ features were relatively simple. We demonstrated that our method achieved a slight improvement of at least 0.1 over SSD and greatly reduced the training time.

## Conclusion

This work adopted ZF Net because its architecture strikes a nice balance in depth: it is deep enough to self-learn the progression of local image features from low- to mid- to high-level, and it is also shallow enough so that the experiments can be conducted quickly. Alternatively, deeper architectures such as VGG, GooleNet, Squeeze-and-Excitation Net and Residual network have been used and have shown relatively good performance in the challenges of computer vision. However, the purpose of this work is not only to achieve high performance on the original images but also to optimize the tradeoff between detection accuracy and processing speed. Moreover, in the real world, datasets are usually limited. To achieve a good performance, original images needed to be processed as multi-scale images. Furthermore, in this work, the effects of some important parameters on the results are discussed, and the best performance parameters were selected.

Our approach can be improved in the future as follows: (1) The training dataset may be augmented, for example, by changing the background of objects; (2) More layers of the model can be added that can help the model learn more features from images; (3) The training model could be improved by training the model with different scaled images under a larger dataset; (4) Identifying different growth processes of wheat mites and evaluating the damage-grade.
